# The chimerical and multifaceted marine acoel *Symsagittifera roscoffensis*: from photosymbiosis to brain regeneration

**DOI:** 10.3389/fmicb.2014.00498

**Published:** 2014-10-02

**Authors:** Xavier Bailly, Laurent Laguerre, Gaëlle Correc, Sam Dupont, Thomas Kurth, Anja Pfannkuchen, Rolf Entzeroth, Ian Probert, Serge Vinogradov, Christophe Lechauve, Marie-José Garet-Delmas, Heinrich Reichert, Volker Hartenstein

**Affiliations:** ^1^Université Pierre et Marie Curie -CNRS, FR2424, Functional Exploration in Marine Model Organisms - Centre de Ressources Biologiques Marines, Station Biologique de RoscoffRoscoff, France; ^2^Université Pierre et Marie Curie -CNRS, UMR 7139, Marine Plants and Biomolecules, Station Biologique de RoscoffRoscoff, France; ^3^Department of Biological and Environmental Sciences, The Sven Lovén Centre for Marine Sciences – Kristineberg, University of Gothenburg – FiskebäckskilSweden; ^4^TU Dresden, DFG-Research Center for Regenerative Therapies DresdenDresden, Germany; ^5^Institute of Zoology, Technical University DresdenDresden, Germany; ^6^Université Pierre et Marie Curie -CNRS, FR2424, RCC (Roscoff Culture Collection) - Centre de Ressources Biologiques Marines, Station Biologique de RoscoffRoscoff, France; ^7^Department of Biochemistry and Molecular Biology, Wayne State University School of MedicineDetroit, France; ^8^INSERM, UMR S 968, CNRS/Université Pierre et Marie Curie - Institut de la Vision/Centre Hospitalier National d'Ophtalmologie des Quinze-VingtsParis, France; ^9^CNRS UMR 7144 and Université Pierre and Marie Curie, EPEP - Evolution of Protists and Pelagic Ecosystems, Station Biologique de RoscoffRoscoff, France; ^10^Biozentrum, University of BaselBasel, Switzerland; ^11^Department of Molecular Cell and Developmental Biology, University of CaliforniaLos Angeles, CA, USA

**Keywords:** acoel, *roscoffensis*, symbiosis, regeneration, brain, algae, *tetraselmis*, model

## Abstract

A remarkable example of biological engineering is the capability of some marine animals to take advantage of photosynthesis by hosting symbiotic algae. This capacity, referred to as photosymbiosis, is based on structural and functional complexes that involve two distantly unrelated organisms. These stable photosymbiotic associations between metazoans and photosynthetic protists play fundamental roles in marine ecology as exemplified by reef communities and their vulnerability to global changes threats. Here we introduce a photosymbiotic tidal acoel flatworm, *Symsagittifera roscoffensis*, and its obligatory green algal photosymbiont, *Tetraselmis convolutae* (Lack of the algal partner invariably results in acoel lethality emphasizing the mandatory nature of the photosymbiotic algae for the animal's survival). Together they form a composite photosymbiotic unit, which can be reared in controlled conditions that provide easy access to key life-cycle events ranging from early embryogenesis through the induction of photosymbiosis in aposymbiotic juveniles to the emergence of a functional “solar-powered” mature stage. Since it is possible to grow both algae and host under precisely controlled culture conditions, it is now possible to design a range of new experimental protocols that address the mechanisms and evolution of photosymbiosis. *S. roscoffensis* thus represents an emerging model system with experimental advantages that complement those of other photosymbiotic species, in particular corals. The basal taxonomic position of *S. roscoffensis* (and acoels in general) also makes it a relevant model for evolutionary studies of development, stem cell biology and regeneration. Finally, it's autotrophic lifestyle and lack of calcification make *S. roscoffensis* a favorable system to study the role of symbiosis in the response of marine organisms to climate change (e.g., ocean warming and acidification). In this article we summarize the state of knowledge of the biology of *S. roscoffensis* and its algal partner from studies dating back over a century, and provide an overview of ongoing research efforts that take advantage of this unique system.

## Introduction

Photosymbiosis represents around 50% of marine photosynthesis, as exemplified by corals and other reef animals as well as by the remarkable biomass and diversity of oceanic photosymbiotic protists (unicellular eukaryotes) from the taxonomic super-groups Rhizaria, Alveolates, and Stramenopiles (Baldauf, 2008). An association between a host (multi- or unicellular) and an “algal” photosymbiont represents, in principle, a “domestication” of photosynthesis that can result in a trophic independence as long as the partners are located in the euphotic zone in view of the infinite source of solar energy. As a consequence, photosymbiosis has a strong global ecological impact and, hence, the adaptations and innovations that contribute to photosymbiotic interactions require both in depth functional and molecular analysis. This analysis is also important since the evolutionary success of photosymbiosis, based on innovative combinations of hosts/photosynthetic symbionts, contributes markedly to marine diversity. Finally, a comprehensive understanding of the mechanisms underlying the recruitment of photosynthetic symbionts by heterotrophic organisms may also contribute to and understanding of the ancestral evolutionary events of acquisition of plastid-like endosymbionts by a primitive heterotrophic eukaryotic cells, i.e., primary and secondary endosymbiosis.

**Figure 1:**
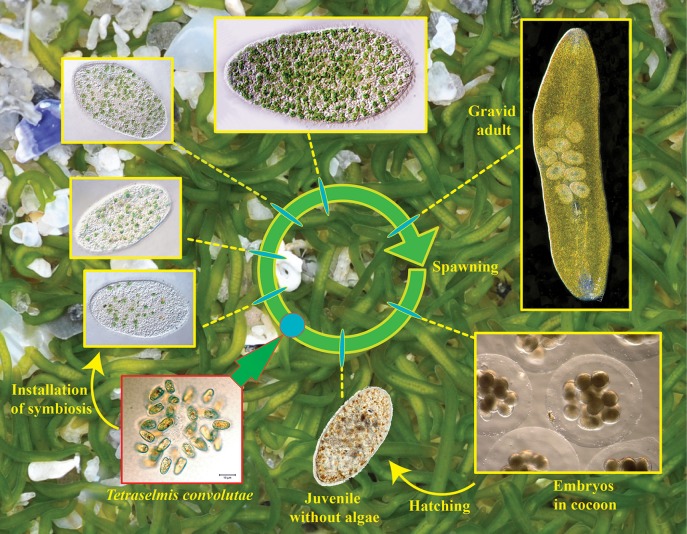
**Life cycle of *Symsagittifera roscoffensis***. In the background minute and elongated adults (around 4 mm length) swimming and gliding upon and inside the sand at low tide.

**Figure 2:**
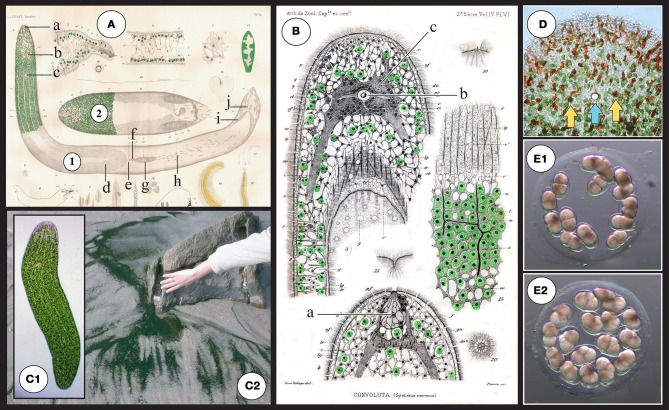
**(A)** Original drawings from Graff (1891) depicting structures of *S. roscoffensis* **(1)** together with a closely related (dwarf) species *S. schultzii* (ex *Convoluta schultzii*) **(2)** and in particular the localization of algae in the epidermal area. **a**: frontal organ (sensing), **b**: statocyst **c**: mouth, **d**: ovocyte, **e**: female genital pore, **f**: bursal nozzle [the bursa nozzle leads from the seminal bursa (g) to the female genital opening (e)], **g**: seminal bursa, **h**: sagittocysts, **i**: seminal vesicles, **j**: male genital pore. **(B)** Original drawings from Delage (1886) **a**: frontal organ (sensing), **b**: (statocyst) **c**: central nervous system (gray area surrounding the statocyst). **(C1)** Adult symbiotic *S. roscoffensis* (see also magnification of the anterior pole in **D**). Note the presence of four visible lines running along the body corresponding to thin longitudinal dorsal and lateral neural bundles. **(C2)** Dark green mat on the beach: a typical colony of *S. roscoffensis* in the residual flows on ebb tides close to Roscoff, Brittany, France. **(D)** Magnification of the anterior part. Note the reduced presence of algae surrounding the anterior pole and the occurrence of reddish structures; these rod-shaped epidermal mucus secretion bodies are called rhabdoids (Smith et al., 1982). Yellow arrows show the two photoreceptors flanking the central statocyst involved in gravity sensing (the blue arrow). (**E)** Egg capsule (cocoons) with early developmental stage embryos **(E1)** 2-cell stage with two macromeres. **(E2)** 4-cell stage: first duet of micromeres.

**Figure 3:**
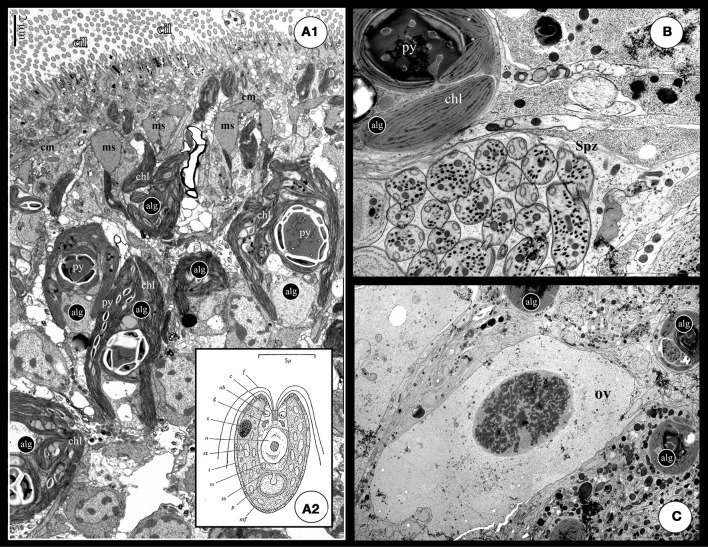
**(A1)** TEM cross section of a *S. roscoffensis* adult. On the top of the image, the ciliated (cilia: cil) epidermis is visible above a net of both circular and longitudinal muscles (cm and lm). Algal cells (alg) are in contact with the acoel cells below the muscles layer. Algal cells exhibit two specific features, the chloroplasts (Chl) and the pyrenoid with starch accumulation on the outside (the white mass). Note some algal extensions close to the epidermis, above the muscle boundary. **(A2)** Detailed scheme of the free-living unicellular green alga, *Tetraselmis convolutae* (modified from Parke and Manton, 1967): c, chloroplast; f, flagellum; g, golgi body; m, mitochondrion; mf, muciferous body; n, nucleus; p, pyrenoid; rb, refractive body of unknown nature; ss, starch shell; s, stigma; st, stroma starch; t, theca. **(B,C)** These two pictures exhibit a clear physical proximity of germinal cells, respectively sperm (Spz in **B**) and ovocyte (ov in **C**), and algae (alg). Such a situation could increase possible horizontal gene transfers.

We introduce here *Symsagittifera roscoffensis* (formerly *Convoluta roscoffensis*), a marine flatworm-like animal that belongs to the phylum Acoela (a = non, coela = cavity), as an ideal photosymbiotic biological model system for exploring the functional biology, molecular biology and evolution of photosymbioses. *S. roscoffensis*, described as a “Plant-Animal” (Keebles, 1910) a century ago, is endemic to the intertidal zone of European coasts of the Atlantic Ocean and the English Channel, and at low tide forms huge colonies composed of millions of individuals (Figures 1, 2A–C). The vivid green color of the colonies is due to the abundant presence of a unicellular green algal endosymbiont, namely the prasinophyte *Tetraselmis convolutae* (Figure 3A). This animal presents a wealth of biological features that are relevant for investigating key phenomena of the photosymbiotic interaction such as the development, genetics, and cell biology of symbiogenesis that are not easily addressed in other more conventional model systems. Importantly, while other experimental systems are well established for the study of some of these aspects, the combination of features exhibited by *S. roscoffensis* and its associated symbionts (and indeed acoels in general) is exceptional in its breadth and diversity, and this makes it possible to address a suite of biological questions that cannot be easily approached in any other single model system. Indeed “to resolve the puzzle of metazoan evolution and development, bioinformatic and experimental approaches must be applied to a wider range of species than just the standard model organisms” (Tessmar-Raible and Arendt, 2004).

From the late nineteenth century onwards, this intriguing green flatworm has been the subject of investigations of the symbiosis between the metazoan host and the unicellular green alga that were focussed mainly on trophic exchanges and energy contribution. An important development for experimentation occurred in the 1960s when the phycologist L. Provasoli (1908–1992, Lehman, 1993), managed to establish laboratory cultures in New York of multiple generations of wild type *S. roscoffensis* originating from the coasts of Brittany, France (Parke and Manton, 1967; Provasoli et al., 1968). However, for various reasons, from 80's onward research on *S. roscoffensis* slowly declined despite the clear potential for addressing key biological issues ranging from photosymbiosis to regeneration, and including development, ecology, and genetics. This lack of experimental progress is especially unfortunate for issues in metazoan evolution. This is because *S. roscoffensis* as an acoel represents something of a Gordian knot in the debate concerning the features of the ancestor of the all bilaterian animals (the so-called “urbilaterian”). In this debate some scientists propose that all Bilateria are descendants of a primitively coelomate ancestor (archicoelomate hypothesis, Salvini-Plawen, 1978; Balavoine, 1998) implying that flatworms have secondarily lost their coelom and are thus not basal bilaterians, while others argue that acoel flatworms are primarily simple and that their acoelomate condition is representative of the ancestor of the Bilateria (planuloid-acoeloid hypothesis, Salvini-Plawen, 1978; Baguna and Riutort, 2004a).

Thus, from a historical point of view, the current renaissance in experimental investigations of *S. roscoffensis* represents a renewal with a rich and relevant background of over a century of studies dedicated to this acoel flatworm from the early 1870s until the 1980s. In this report, we review the current state of knowledge concerning the biology, ecology, and physiology of this animal, and we highlight the many new avenues of research made possible by the precise experimental control of the ontogeny of this marine flatworm including induced symbiogenesis through co-culture of the acoel and the algal symbiont.

## Historical background on *S. roscoffensis* and its symbiont

Studies beginning in the late nineteenth century centered on the origin and the role of the enigmatic “green cells” inhabiting the body of *S. roscoffensis* (thought to be chloroplasts because of starch accumulation and oxygen production) (Geddes, 1879; Delage, 1886). Initially, *S. roscoffensis* was mistakenly referred to *Convoluta schultzii* a similar green acoel species previously described from the Adriatic sea (Schmidt, 1852). Shortly afterwards, however, a formal re-examination of the acoel species from Brittany showed clear morphological differences and it was named *Convoluta roscoffensis* because of its abundance in the vicinity of the Station Biologique de Roscoff (Graff, 1891; Graff and Haberlandt, 1891) (Figure 2). The enigmatic green photosynthetic corpuscles (“zoochlorellae”) were later unambiguously ascribed to the algae in the detailed studies of Keebles and Gamble (1905, 1907). These authors documented their substantial set of original experiments and observations on the biology, ecology and behavior of *S. roscoffensis* and the associated symbiosis in a book entitled “Plant animals, a study in symbiosis” (Keebles, 1910). The symbiotic quadriflagellate green alga, formally described and named *Platymonas convolutae* (Parke and Manton, 1967), was characterized further by experiments on culture conditions and the initiation of the symbiosis (Provasoli et al., 1968). Subsequently, *Platymonas convolutae* was renamed *Tetraselmis convolutae* following a detailed structural study of the genera *Tetraselmis*, *Platymonas*, and *Prasinocladus* which led to the conclusion that species in these genera should be united in the same genus, *Tetraselmis* (Norris et al., 1980). Still later, a detailed histological study of the acoel led to reclassification of *Convoluta roscoffensis* as *Symsagittifera roscoffensis* (Kostenko and Mamkaev, 1990).

## *Incertae sedis*: the controversy concerning acoels as extant basal bilaterians

Delage (1886) was one of the first zoologists to discuss the “inferiority of organization” and the relationship of *S. roscoffensis* to other animals. Numerous studies over more than a century involving cytological, morphological, physiological, and genetic approaches have produced contradictory results and hypotheses concerning the phylogenetic position of acoels within the metazoans. Traditionally affiliated with the Platyhelminthes (Hyman, 1951; Karling, 1974; Ehlers, 1985; Ax, 1996), several comparative morphological and/or molecular analyses have suggested that acoels are not members of this phylum (Ruiz-Trillo et al., 1999, 2004), but rather that together with the Nemertodermatida they form the phylum Acoelomorpha (Baguna and Riutort, 2004b) which is located at the base of the Bilateria (Ruiz-Trillo et al., 2002; Pasquinelli et al., 2003; Telford et al., 2003; Cook et al., 2004; Glenner et al., 2004; Garcia-Fernandez, 2005; Jimenez-Guri et al., 2006; Sempere et al., 2007). Later, the phylum Acoelomorpha was dismissed as paraphyletic, with acoels and nemertodermatids considered separate early bilaterian clades (Wallberg et al., 2007). However, the debate is still ongoing as illustrated by recent investigations which raised objections to Deutsch (2008) or revisited (Philippe et al., 2007) the systematic position of acoels at the base of the Bilateria.

In recent molecular genetic reconstructions that employed broad genomic sampling in an attempt to resolve the animal tree of life, the phylogenetic position of the Acoela was found to be unstable (Dunn et al., 2008). Indeed it has even been shown that some developmental (gene expression patterns) and morphological characters (related to stem-cells) are shared, apparently synapomorphically, between acoels (specifically the acoel *Isodiametra pulchra* and Platyhelminthes (specifically *Macrostomum lignano*) (Egger et al., 2009). The difficulty to phylogenetically stabilize acoels in the metazoan tree of life is illustrated by two independent large-scale metazoan phylogenomic surveys including transcriptomic data from 3 and 4 acoels respectively, including *S. roscoffensis*. In one of these surveys, these acoel flatworms form a sister clade to all other bilaterian animals (Hejnol et al., 2009). In the other survey, acoels are deuterostomes related to Xenotubella, forming Xenacoelomorpha (Philippe et al., 2011), a sister group of the Ambulacraria (hemichordates and echinoderms).

## The biology of *S. roscoffensis*

“Imagine a minute, elongated fragment of a most delicate leaf, some 1/8 in. long by 1/16 in. broad, and you have a picture of *C. roscoffensis*. Imagine, further, myriads of such green, filmy fragments lying motionless on moist, glistening patches of a sunny beach between tide marks and you see the species in its native habitat” (Keebles, 1910).

*S. roscoffensis* is a gregarious intertidal symbiotic acoel endemic to the North Atlantic coasts. Around 4 mm in length, it lives interstitially and is found during daylight on the upper part of beaches in calm residual flows on ebb tides. The number of algal cells within adult *S. roscoffensis* individuals has been estimated to be around 40,000 (Doonan and Gooday, 1982). Up to millions of individuals congregate and form mats in more or less discrete patches notably where exposed to the sunlight that favors photosynthetic activity of the photosymbiotic algae (Figures 1, 2). At high tide animals retreat into the sediment to escape dispersal by waves. Worms are surrounded by a viscous mucous layer that they abundantly secrete (Fraenkel, 1961) and which is assumed to aid locomotion by ciliary gliding and to allow adhesion (Martin, 2005). Our observations based on flow-cytometry measurements suggest that this biofilm is also a crucial interface that harbors a specific bacterial community which may be a third and essential partner in a complex symbiotic relationship.

### Morphology

*S. roscoffensis* is a hermaphroditic, soft-bodied, longitudinally curved worm with a syncytial gut that opens at the ventral surface. A mesoderm-lined body cavity (coelom) is absent. The body wall consists of multi-ciliated epidermal cells with interspersed glands and an irregular layer of muscle cells. Animals move by ciliary action. The nervous system consists of a central ganglion (brain) that surrounds a statocyst (gravity sensor) and a pair of rhabdomeric eyes. A characteristic feature of the taxon Sagittiferidae (Mamkaev and Kostenko, 1991) is the sagittocyst, a capsule-like extrusome containing a protrusible needle-like structure that is physically linked to the copulatory organs.

### Development

During the breeding period from September to June (Douglas, 1983b), *S. roscoffensis* lay up to 20–30 eggs per sexually mature individual, confined in a translucent mucilaginous egg capsule (cocoon) (Figure 1). As observed for other acoel species, embryos in *S. roscoffensis* are discharged through breaks in the body wall. Release of embryos and elaboration of the cocoon are concomitant. The formation of the cocoon has been clearly documented (Costello and Costello, 1939; Apelt, 1969) to occur through a rotation of the worm end over end coupled with the secretion of a jelly-like substance in which both the worm and the embryos are initially enclosed. Subsequently, the animal usually works its way out of the cocoon leaving the embryos behind. In some cases active symbiotic adults have been found enclosed in the cocoon together with the developing embryos, however, this may represent an artifact of culturing conditions as such since comparable observations have never been made in natural environment (Apelt, 1969). When wild type animals are recovered from the shore and transferred into lab conditions, *S. roscoffensis* begins to deposit fertilized eggs (cocoons) after 4–5 days, when larger clusters containing uncleaved or 2 cells stage embryos can be recovered daily. In the wild, embryonic development lasts 3–5 days before the aposymbiotic juveniles hatch and swim freely in the water column.

**Figure 4:**
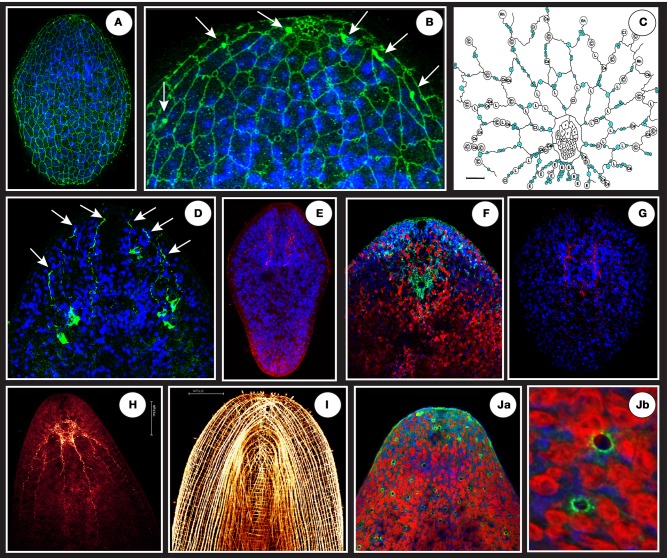
**(A)** A net-like structure labeled by a VDAC antibody (in green) covering the epidermal surface of a juvenile connecting putative monociliated sensory receptors and gland openings. **(B)** The circles appearing on the magnification (focus on anterior tip = “the head”) are schematically represented in **(C)** (modified from Smith and Tyler, 1986: blue circles are sensory receptors, other circles represent different gland types openings). Note at the top of the anterior tip in B a dense and honeycombed-like structure corresponding to frontal gland extremity tip. The white arrows in **(B)** shows 3 dense and localized signals flanking the frontal gland that are ends of projections coming from a deeper cell body-like structure shown in **(D)**. **(E)** A specific signal (in red) using ATPsynthase human antibody localized in the brain area in a juvenile. **(F)** Anterior tip of an adult showing in green (surrounding the statocyst) the specific signal of *S. roscoffensis* neuroglobin co-localized with brain (blue is Dapi and Red auto-fluorescence of algae). **(G)** A specific signal (in red) using glial cell directed human antibody (GFAP) localized in the brain area of a juvenile. **(H)** Adult RFaminergic nervous system. **(I)** Phalloïdin staining showing longitudinal, circular, and transversal muscle fibers. **(Ja,Jb)** Human P53 (tumor suppressor) antibody reveals dispersed and non-ordered gland-like openings on the surface.

Under laboratory conditions, embryonic development lasts for 4–5 days and leads to the hatching of colorless juveniles (larvae). During the first day, the fertilized egg undergoes a series of divisions following a pattern called duet spiral cleavage (Boyer, 1971; Henry et al., 2000) in contrast to the more widespread quartet spiral cleavage found in many lophotrochozoans (Giribet, 2002). Cleavage results in a solid stereroblastula, whose inner cells soon merge into the syncytial gut, while the outer cells represent the primordium of the body wall and associated nervous system. During the second half of embryogenesis, the ciliated epidermis at the surface differentiates and becomes separated from the interior primordia of the brain and muscle layer (Figures 4H,I). Neurons form longitudinal cords and commissures during day 4, at the same time when muscle fibers establish their orthogonal pattern underneath the epidermis.

### Ecology: acquisition of algal symbionts

Juveniles ingest environmental free algae within the first few days after hatching. Algae are taken up into a vacuole in the syncytial central parenchyme where they lose their cell wall (theca), the four flagella and the eyespot (light receptor), resulting in a significantly modified phenotype (Oschman, 1966; Provasoli et al., 1968; Douglas, 1983a). To date, the sequence of events leading to an algal protoplast after vacuolization is unknown: specific enzymatic animal digestion? Regression of the algal structures via gene repression? Modified algae are released from the central syncytium into the extracellular spaces of the parenchyma. Algae are extracellular (i.e., not integrated in the animal cells) (Oschman and Gray, 1965) and rapidly divide mitotically and become associated with the body-wall, extending chloroplast-filled projections that intercalate with muscle fibers and epidermal cells (Figures 3A1,A2). It is likely that the membrane surrounding the algae is similar to symbiosome membranes found in dinoflagelate/cnidarian photosymbiosis with vital signaling and trafficking properties. We have isolated and cultured clonal strains of *T. convolutae*, directly recovered one by one from crushed *S. roscoffensis* adults between microscope slides and coverslip and then transferred in a specific culture media. Once free, the symbiotic algae recovers cell, flagella and the eyespot.

Colorless aposymbiotic juveniles must encounter free-living *T. convolutae* cells in order to initiate the obligate symbiosis and develop directly without intermediate larval stages. If present, they can ingest algae naturally occurring in seawater otherwise algae can also be supplied from controlled laboratory cultures. After natural or artificial induction of symbiosis, aposymbiotic juveniles become fully green within 10–15 days (Douglas, 1983a), whereas worms that remain aposymbiotic decline progressively and do not survive for more than 20 days. *S. roscoffensis* can withstand a large range of temperature in culture (roughly from 10°C or less to 20°C), reflecting the annual temperature range in its natural habitat. When cultures were initially established in New York, Provasoli obtained cocoons after 45–60 days following egg laying, a period that thus represents the generation time for cultured *S. roscoffensis*. Additional observations are needed during this 45–60 day interval to clearly identify the stage at which gonads become mature. In any case, growth and gonadal maturation are strictly dependent on symbiogenesis.

### Ecology: trophic relationships between the two (or possibly three) partners

In the 70's, metabolic profiles that attempted to explain the establishment of this photosymbiosis were mainly deduced from the combination of (1) the use of radioisotopes, isotopic tracer experiments and chromatography techniques and (2) the ability of cultivating independently symbiotic adults, aposymbiotic juveniles and algal strains. Captive algae produce photosynthetates, such as mannitol and glutamic acid, which are at the origin of the synthesis/release of amino acids by the worm (Boyle and Smith, 1975). Algal lactic acid also represents a source of nutrients for *S. roscoffensis*. Lactate, which is a major component of extracellular exudates of *T. convolutae*, has been shown to be metabolized into amino acids when supplied exogenously to aposymbiotic juveniles (Taylor, 1974). *S. roscoffensis* individuals do not de novo synthesize long chain saturated/unsaturated fatty acids and sterols, but rather depend directly on biosynthesis and supply from the algal symbiont (Meyer et al., 1979).

On hatching, juveniles exhibit solid uric acid that increases during the aposymbiotic state but decreases gradually to undetectable levels once the association with the algae take place (Douglas, 1983c). The endogenous uric acid, a result of the host nitrogen waste products, represents an important source of nitrogen for *T. convolutae* of which recycling and consumption is correlated with photosynthetic activity (Boyle and Smith, 1975; Douglas, 1983c). As demonstrated for corals, recycled inorganic nitrogen is a major process in the zooxanthellae's nitrogen demand (Rahav et al., 1989).

The active uptake of nitrates by *S. roscoffensis* adults depending on light availability (10 times higher than that of its symbiont alone) (Carvalho et al., 2013) is in agreement with primary productivity by coral zooxanthellae which is about an order of magnitude higher than that of free-living phytoplankton on reefs (Muscatine, 1980). *S. roscoffensis* is thought to be involved in the uptake of nutrient rich submarine groundwater discharge seepage, in other words may be a possible nitrate recycler (Carvalho et al., 2013).

A key metabolical feature of the photosymbiotic entity acoel/zooxanthella is the presence of dimethylsulphoniopropionate (DMSP). DMSP is produced by the photosymbiont and found in high concentration in the host together with associated enzymatic breakdown products acrylic acid and the volatile gas DMS (Dimethylsulphide) a sulphated compound massively generated into the host environment. DMSP may confer specific advantages to the photosymbiotic acoel and is thought to be a be multifunctional defense precursor (Wolfe et al., 1997), which acts as an efficient predator repellent. Interestingly, recent work has shown that photosynthetic organisms are not the only producers of DMSP demonstrating that coral juveniles lacking photosynthetic symbionts can produce DMSP under normal or stressed conditions (Raina et al., 2013).

Cultures of the DMSP producing algal photosymbiont do not show DMSP-lyase activity (Van Bergeijk and Stal, 2001). This enzymantic lyase activity might be constitutively expressed by the acoel. Alternatively, it might be generated by the bacterial cohort that naturally surrounds *S. roscoffensis*, and if this is the case the symbiotic unit would comprise an elaborated “ménage à trois,” the host, the photosymbiont and a bacterial consortium hosted in the host's mucus secretions acting as an active biofilm (Ducklow and Mitchell, 1979).

### Ecology: symbiont selection and ingestion

Despite the fact that maternal transmission of algal symbionts has been described in another acoel species (Barneah et al., 2007), strict horizontal transmission occurs in *S. roscoffensis* (Douglas and Gooday, 1982) which indicates highly specific (and unknown) mechanisms of recognition and selection of one or a restricted set of algal species among the hundreds if not thousands present in coastal seawater. Provasoli experimentally demonstrated a hierarchical order in competitive efficiency among different algal taxa (including the true symbiont) that he tested *in vitro* for inducing photosymbiosis. Surprisingly, he also found that a “less effective algal symbiont which had already colonized a larva was found to be actively replaced by a more effective one admitted later” (Provasoli et al., 1968). Expulsion of an alien algae “via the normal mucilage ducts or other temporary or permanents opening” (Provasoli et al., 1968) and replacement is a well-known mechanism in cnidarian (corals) and may also be a possible mechanism for the regulation of symbiont density in the acoel (Fishman et al., 2008).

As shown for *S. roscoffensis* from south Wales (Mettam, 1979), two (biochemically distinct) subgenera of the genera *Tetraselmis* (Hori et al., 1982), namely *Tetraselmis* (ex *Platymonas*) and *Prasinocladia*, can be present in the host, but never mixed together in the same specimen (McFarlane, 1982a). While only 20–45% of *S. roscoffensis* from the south Wales cohort contained *Tetraselmis*, populations from coast of France and the nearby Channel Islands mainly form associations with the subgenus *Tetraselmis* (ex *Platymonas*) (McFarlane, 1982b). To explain this apparent discrepancy, Douglas (1985) hypothesized a possible higher mortality at an uninvestigated stage in the life-cycle or a failure of juveniles to encounter *Tetraselmis* in south Wales (*Tetraselmis* could have been less common than *Prasinocladia* in this area).

Finally, it should be mentioned that the exact status of the symbioses in acoels hosting algae is still a subject of debate. The mutualistic nature of the *S. roscoffensis*/*T. convolutae* association has been challenged since it has not been shown whether, under natural conditions, algal cells confined in the acoel are able to survive and reproduce after death of the host. In other words, it is still possible that the confinement of the algae in the acoel is actually a sequestration and unilateral misappropriation of photosynthetic activity by the host and thus an evolutionary *cul-de-sac* for the algae (Selosse, 2000).

## A multifaceted biological system

In a comprehensive review dealing with algal symbiosis in flatworms, McCoy and Balzer (2002) underlined “the growing recognition of the importance and prevalence of flatworm-algal symbioses worldwide. Together with the inherent advantages of these associations for laboratory studies, these signs suggest the near future may hold great revelations within the field of flatworm-algal interactions, and thus to symbiosis as a whole.” Indeed, the remarkable potential of *S. roscoffensis* for exploring the many largely unstudied, diverse and multifaceted aspects of marine symbiotic systems is currently becoming apparent.

### An emerging model system for exploring marine photosymbiosis

Detailed insight into the mechanisms that operate in marine photosymbiotic systems is still largely lacking. While intensive experimental investigations have been carried out on photosymbiotic corals and anemones (cnidarian/dinoflagelate associations) due to their ecological importance for coral reef ecosystems, the difficulty of independently culturing the corresponding symbiotic partners, the complex life cycles as well as cross-contaminations have been significant limiting factors for this research area. For example, in most cases little is known about the molecular bases of chemotaxis, specific partner recognition and selection, the metabolic relationships between a heterotrophic and a photosynthetic organism, or the role of potential gene transfer between symbiont and host especially in the eukaryote/eukaryote situation. These issues can be addressed experimentally in the *S. roscoffensis* model system notably due to the easy access to the four phases of the symbiosis, namely embryogenesis, aposymbiotic alga, aposymbiotic juvenile and functional photosymbiotic entity. Importantly, the *in vitro* control of symbiosis induction in the *S. roscoffensis*/*T. convolutae* biological system allows precise characterization of the dynamic transcriptional and translational adjustments that occur between the two partners during the ontogenetic acquisition of photosymbiosis during ontogenesis and of the associated adaptations that are required for the maintenance of such a partnership.

The *S. roscoffensis*/*T. convolutae* system will help to address problems of marine photosymbioses such as the bleaching phenomena and other causes for the breakdown of symbiosis (Venn et al., 2008). More than 80 years ago Drzewina and Bohn pioneered experimental studies showing that the acidification of seawater caused *S. roscoffensis* to eject most of its algae and die (Drzewina and Bohn, 1924). Employing comparable experimental techniques, later studies (Boyle and Smith, 1975) demonstrated that at least half of the carbon fixed in photosynthesis moved from algae to animal. While acoels have mainly been described for littoral marine habitats, pelagic acoels are likely to be ecologically significant in open oceans and may represent an unsuspected source of primary productivity through carbon-fixing photosymbioses with algae (Stoecker et al., 1989).

Finally, a key aspect of photosymbiosis is the possible occurrence of lateral gene transfer from the symbiont to the host. Although the exchange of genetic material between metazoans and symbionts is thought to be very rare, the obligate symbiosis of *S. roscoffensis* might involve such gene transfer. The algae cells in the acoel are physically close to spermatozoid-containing vacuoles (follicular testis) enhancing the possibility and probability for integrating segments of algal DNA into the acoel germline (Figures 3B,C).

### A model for a mechanistic understanding of the impact of global climate changes

The Intergovernmental Panel on Climate Change recently published its report on the impacts of climate change (IPCC 2014) and concluded that it is “*virtually certain*” that human influence through CO_2_ emissions has warmed the ocean and led to significant changes in ocean chemistry resulting in an increased seawater acidity known as ocean acidification. Investigations of the consequences for marine organisms and ecosystems have a relatively short history and led to conflicting results. The IPCC concludes that “*a pattern of positive and negative impacts emerges (high confidence) but key uncertainties remain in our understanding of the impacts on organisms*.” In this respect, predictions of the effects on ecosystems remain problematic and a major scientific bottleneck and key conceptual gap is the lack of knowledge of the mechanisms and overarching principles for how environmental changes affect organisms (Dupont and Pörtner, 2013).

Many marine species predicted to be highly sensitive to global changes are photosymbiotic, including foraminifera and corals. Foraminifera are a group of unicellular zooplankton forming chambered calcite shells hosting symbiotic algae. The overall evidence strongly suggests that ocean acidification will have a significant effect on planktonic foraminifera including significant impact upon calcification (Lombard et al., 2010). Risks to tropical coral reefs are also of great concern, since the livelihoods of around 400 million people depend on such habitats. Tropical coral reef ecosystems represent one of the most diverse habitats in the oceans, being home to about a third of all marine species. Many studies demonstrate a reduction in growth (net calcification rates) in response to ocean acidification. However, this is not an ubiquitous response, with different species exhibiting negative (Ohde and Hossain, 2004), no measureable response (Reynaud et al., 2003), or variable responses (Gattuso et al., 1998) to reduced ocean acidification. Thus, major questions remain notably how and why coral are sensitive to ocean acidification, and these are the subject of recent research initiatives that investigate the mechanism of calcification.

The question of the direct impact of global stressors on photosymbiotic relationships between photosynthetic and non-photosynthetic organisms remains unclear. The symbiosis is regulated by intrinsic and extrinsic parameters and can break down, a phenomenon known as bleaching, under certain conditions including ocean warming and acidification (Yellowlees et al., 2008). However, the factors that are directly responsible for bleaching are poorly understood and investigations are complicated by other physiological processes that are known to be sensitive to these stressors including calcification (Gattuso et al., 1998) and feeding (Stumpp et al., 2013).

*S. roscoffensis* and its endosymbiont *T. convolutae* constitute an alternative model for a mechanistic understanding of the direct impact of global changes on photosymbiosis. Its biology allows studying photosymbiosis without any interference from calcification or heterotrophic feeding. Moreover, it is also possible to study the stressors' impact on algae and worms individually (pre-symbiosis juvenile stage). In stricking contrast with other studied photosymbiotic species such as corals or foraminifera, *S. roscoffensis* has shown a strong resilience to ocean acidification with no negative effects on fitness and/or photosymbiotic relationship at pH values down to 6.0 (Dupont et al., 2012). This result indicates that photosymbiosis can be resistant to ocean acidification and that the observed negative effects on foraminifera and corals could occur through indirect impacts at other levels (feeding, calcification). Preliminary results on oxygen production and respiration (Dupont, personal communication) show that the acoel responds to an exposure to decreased pH through an increased respiration, a phenomenon observed in many other invertebrates (Dorey et al., 2013). This can reflect additional energy costs, e.g., for acid-base regulation (Stumpp et al., 2012). When kept in the dark, both algae alone and the worm with the algae can maintain their respiration constant until a tipping point at pH 6.3 leading to a metabolic depression. In presence of light, algae are also able to increase their oxygen production with decreasing pH until a tipping point at pH 6.0. These results suggest that the algal partner is the “weakest link” in the symbiosis and driving the response to ocean acidification.

### A model system for studying the organization of a basal brain

Classical studies of the acoel central nervous system, some of them based on *S. roscoffensis* (Delage, 1886; Graff and Haberlandt, 1891), have described a bilobed central ganglionic brain that possesses many neuronal cell bodies and a complex pattern of commissural and connective fiber bundles. In contrast, subsequent, immunocytochemical studies using antibodies against neurotransmitters came to the conclusion that the acoelomorph central nervous system should be more aptly described as a relatively simple “commissural brain,” consisting of an anterior aggregate of transverse and longitudinal fibers (Reuter et al., 1998; Gaerber et al., 2007; Kotikova and Raikova, 2008). However, since these types of immunolabeling studies display only small neuronal subsets and do not reveal the nervous system in its entirety, they are not very well suited to reveal comprehensive neuroarchitectural features.

Recently, global antibody markers against neurons combined with serial transmission electron microscopy and 3D reconstruction techniques were applied to study the developing and adult brain of *S. roscoffensis* (Bery et al., 2010; Semmler et al., 2010). These investigations, which focused strongly on the brain of the juvenile (freshly hatched) animal, confirmed early histological findings, demonstrating the presence of a compact anterior brain containing approximately 700 neuronal cell bodies arranged around a central neuropile domain. The neuropile surrounds a central statocyst, and is flanked by two small pigment-cup eyes. Labeling with anti-tyrosinated tubulin demonstrated three pairs of longitudinal nerve cords, which are cross-connected by numerous commissures; nerve cords are flanked by regularly spaced neural cell bodies (Figure 4H).

Other antibody markers have revealed additional details of the *S. roscoffensis* brain. Anti-FMRFamide reveals large clusters of cells in the lateral brain, and the lateral cord as well as brain neurons that send long processes to the anterior tip of the animal, which is known to possess densely spaced arrays of sensory receptors. An antibody raised against the *S. roscoffensis* neuroglobin (Lechauve et al., 2013) protein strongly labels neurons grouped around the statocyst in the center of the brain (Figure 4F). Immunolabeling using human mitochondrial antibodies reveals subsets of cells that are likely connected with nervous system. Thus, the VDAC (Voltage-Dependent Anion Channels, a class of porin ion channel located on the outer mitochondrial membrane) antibody shows two types of immunolabeling. One labels a net that occupies all the surface of the animal and reveals both putative monociliated sensory receptors and gland openings surrounding the frontal gland Smith and Tyler (1986) (Figures 4A–C). The second, a more internal signal, labels sets of neuron-like structures that send projections on each side of the frontal gland (Figure 4D). An other unexpected set of openings is also revealed on the surface of *S. roscoffensis* using the human P53 tumor suppressor antibody as shown in Figures 4Ja,Jb. An ATP synthase antibody labels cells that are in part comparable to those of the serotonergic or RFaminergic nervous system (Semmler et al., 2010) (Figure 4E). A human GFAP (Glial Fibrillary Acidic Protein) antibody shows a specific signal in the brain region (Figure 4G) which may label glia-like structures in the central neuropile and nerve cord. (It must be mentioned that while these cross-reactions with human antibodies may be useful for the identification of new putative structures, they do not imply that the antibodies bind homologous proteins.)

Currently, these immunohistochemical studies are being complemented by a serial transmission electron microscopy project focused on the juvenile *S. roscoffensis* brain. Using the software platform TrakEM2 (Cardona et al., 2012), close to 1000 contiguous sections of the juvenile head are digitally recorded and aligned. Profiles of cells, axons and synapses are segmented manually, and neuronal circuits contained within the sectioned volume (which includes the entire brain) can be reconstructed. This project should provide a wealth of information about neuroarchitectural aspects of the acoel brain, such as axon-dendrite relationships, branch lengths and spacing, and synapse patterns.

### An experimental system for analyzing regeneration

A recent review dedicated to regeneration in plathyhelminths and acoel flatworms highlighted that “old questions and many of the most intriguing phenomena that have been discovered cannot be explained today. Is regeneration recapitulating pathways used in embryonic and postembryonic development? Are stem cells in adult flatworms totipotent, and can they be linked to embryonic blastomeres?” (Egger et al., 2007). The authors concluded that regeneration capacity and the existence of possibly totipotent stem cells in adults are two key features of many flatworms not found in this combination in any other bilaterian taxon. Indeed, acoels are currently considered as highly relevant models for stem-cell research since they have somatic stem cells that are very active in postembryonic developmental processes such as tissue renewal, growth, repair, and regeneration (Bely and Sikes, 2010). Regenerative replacement and reparation of lost body parts remain a challenge in modern medicine and biology, and simple animal models with marked muscle or nervous system regeneration capacities will be useful for characterizing the basal regenerative mechanisms that may in part also operate in complex body plans including those of vertebrates.

Natural spontaneous regeneration following tissue disruption due to egg laying were observed in acoels 70 years ago (Costello and Costello, 1939). In an experimental context, their strong regeneration capacity (and the presence of stem cells) following controlled amputations or incisions make acoels a good model for exploring the underlying developmental and morphogenetic mechanisms. Moreover, additional techniques like transplantation and grafting, of the statocyst for instance (Hanson, 1967), demonstrate the utility of acoels for investigating regeneration in broader contexts (Steinböck, 1963). Finally, in the context of nervous system regeneration and development, *S. roscoffensis* may potentially represents an alternative model for addressing the regenerative properties of glial cells, the most abundant cell type in the mammalian nervous system (Freeman and Doherty, 2006), but not present in all bilaterian phyla (Hartline, 2011).

**Figure 5:**
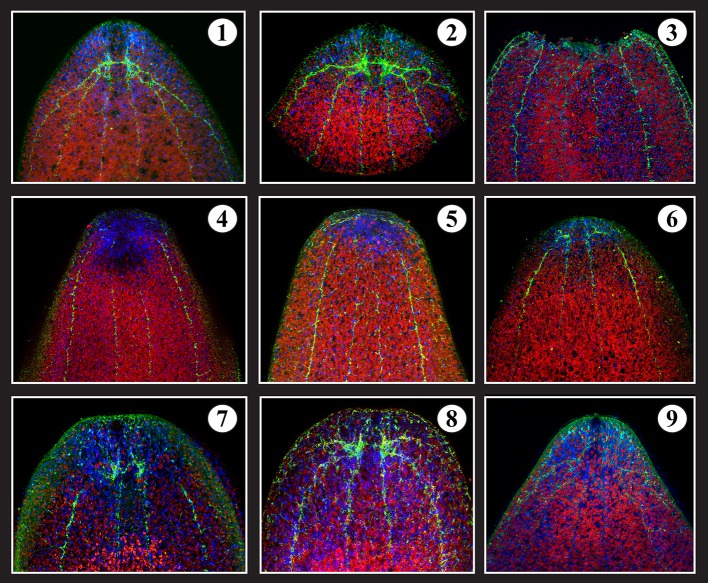
**Brain regeneration**. **1-** Intact serotonergic head (brain) of *S. roscoffensis* (red: algae auto-fluorescence; blue: Dapi; Green: serotonin antibody). **2-** Amputated head. **3-** Decapitated body = T0. **4-** T0 +3 days after amputation. **5-** T0 +6 days. **6-** T0 +10 days. **7-** T0 + 15 days. **8-** T0 + 20 days. **9-** 24 days: complete brain with frontal organ associated with normal behavior.

*S. roscoffensis* exhibits regenerative capacities of both posterior and anterior body parts. After amputation of the head (Figures 5-1–5-3), animals initially show highly aberrant patterns of movement. Having lost anteriorly located chemosensory and mechanosensory receptors, amputated animals swim abnormally straight, rather than showing the swaying (“sniffing”) motions characteristic for intact animals, and this forward swimming is sometimes interrupted by abnormal “forward rolls.” Loss of the statocyst, comprised in the amputated brain, is reflected by the inability of animals to swim downward when taken up by a pipette (normally a remnant reflex of protection mimicking their retreat inside sand). However, within 20–25 days, normal behavior fully recovers, indicating that all or most of the neuronal elements of the brain have been regenerated.

The regeneration process can be directly followed by labeling amputates with markers specific for different types of neurons, such as anti-5 hydroxytryptamine (5-HT; Figure 5). Anti-5HT labels a small set of interneurons with long processes interconnecting the brain hemispheres, and projecting into longitudinal nerve cords (Figure 5-1). Following head/brain amputation (Figure 5-3), the body wall at the wound surface contracts within 1–2 days (Figure 5-4) and the wound area is populated by a dense blastema (Figures 5-4,5-5). The cut ends of the 5-HT-positive nerve cords, which persist after the amputation, are drawn closer to each other during the wound contraction (Figures 5-4,5-5). At around 10 days after amputation, the first newly generated 5 HT-positive neurons appear in the blastema, where they establish commissural tracts between the longitudinal cords (Figures 5-6,5-7). Around 20–24 days after amputation, the brain has been fully restored (Figures 5-8,5-9).

## Concluding remarks

Non-model organisms are generally prone to an uncertain fate in biological science until reliable protocols are developed for a transfer from field to laboratory. Continuous laboratory cultivation and *in vitro* rearing under artificial conditions often represent major challenges: it enables to have access to precise windows of the developmental program from the first cleavage to the death of the model. Several new animal models have recently emerged for exploring metazoan diversity, for investigating the origin of body plans, and for tracing metazoan evolution and other crucial life history traits. Here, we have stressed diverse fields of research that are being investigated or could potentially be investigated using *S. roscoffensis*. Specific features of *S. roscoffensis* could be useful and complementary for the analysis of more general phenomena such as the molecular basis of the mechanisms of photosymbiosis: the easy handling of *S. roscoffensis* and the culture of its photosymbiotic partner represents an emerging model opening new designs of experimental protocols for alternatively questioning endosymbiosis or attain a more concrete idea of reef ecology and the fragility of photosymbioses that face major environmental changes which result in phenomena such as mass bleaching of the emblematic photosymbiotic coral. We also report the potential of employing a combination of approaches for exploring the molecular and physiological basis of other processes such as brain regeneration and the potential of glial cells-like in neural development, function and health but also the superposition of circadian/circatidal rhythms in the field of chronobiology (Tessmar-Raible et al., 2011), these being crucial phenomena in many marine organisms which have been rarely considered in the classical literature (Martin, 1907).

### Conflict of interest statement

The authors declare that the research was conducted in the absence of any commercial or financial relationships that could be construed as a potential conflict of interest.
